# Supplementary material for: Electrochemically active bacteria sense electrode potentials for regulating catabolic pathways
Source: Nat Commun. 2018 Mar 14;9:1083. doi: 10.1038/s41467-018-03416-4 (PMC5852097; doi:10.1038/s41467-018-03416-4)
Supplement: Supplementary file 1 — Supplementary Information [file 41467_2018_3416_MOESM1_ESM.docx]

Electrochemically active bacteria sense electrode potentials for regulating catabolic pathways

Hirose *et al.*

**Supplementary Figure 1** Shifts in current generation by WT and ∆*arcS* cells under potential-controlled conditions. Cells were cultivated in EC containing lactate minimal medium (LMM) supplemented with 10 mM lactate as the electron donor. The working electrode was initially poised at +0.2 V before changing to +0.5 V or –0.1 V. The arrows indicate the time points at which the electrode potential was shifted. Reproducibility was examined in at least three independent trials, and representative data are shown.

**Supplementary Figure 2** Comparison of transcriptional changes by microarray and qRT-PCR analyses. Log_2_-transformed fold changes (log_2_FC) in the expression levels of selected electrode potential-responsive genes determined by qRT-PCR were plotted against those determined by microarray analysis.

**Supplementary Figure 3** Frequencies of genes assigned to each COG category in MR-1 electrode potential-responsive genes. Values are normalized to the COG frequencies of all MR-1 genes.

**Supplementary Figure 4** Comparison of the maximum current density between WT and ∆*cymA* in pyruvate-fed ECs. The working electrode was poised at +0.5 V (HP) or 0 V (LP). Bars and error bars represent means and s.d., respectively (n=3 biological replicates). The values for WT are the same as those shown in Fig. 3.

**Supplementary Figure 5** Intracellular NADH/NAD^+^ ratios in WT and ∆NDH cells exposed to different electrode potentials. The working electrode was poised at +0.5 V (HP), +0.2 V (MP), or 0 V (LP). Bars and error bars represent means and s.d., respectively (*n*=3 biological replicates).

**Supplementary Figure 6** Expression pattern of *nuoI* in WT and ∆*arcS* as determined by qRT-PCR. Bars and error bars represent means and s.d., respectively (*n*=3 biological replicates). Asterisks indicate a statistically significant difference (*P* < 0.05; one-way ANOVA followed by LSD test).

**Supplementary Figure 7** Hierarchical clustering of expression patterns of 322 electrode potential-responsive genes in MR-1. Colors indicate the normalized signal intensities of the microarray data for WT and ∆*arcS* cells grown under high potential (+0.5 V) and low potential (–0.1 V) conditions.

**Supplementary Figure 8** SDS-PAGE of N-his-ArcA protein samples. Protein samples (5 µg) were analyzed on 12.5% SDS-polyacrylamide gels. Lane 1, *Escherichia coli* BL21 (DE3) (pET-arcA) crude extract; lane 2, purified N-his-ArcA; lane 3, molecular weight marker.

**Supplementary Figure 9** Electrophoretic mobility shift assay of ArcA and the upstream regions of *sucA* and *atpI*. Probes containing the upstream regions of SO_0011 and *nuoA* were used as negative and positive controls, respectively. DNA-binding reactions were performed in the presence (+) or absence (–) of 300 ng purified ArcA-P.

**Supplementary Table 1** Bacterial strains used in this study.

| Strain or plasmid | Relevant characteristic | Source or reference |
| --- | --- | --- |
| Bacterial strains |  |  |
| *Escherichia coli* |  |  |
| WM6026 | Donor strain for conjugation; *lacI*^q^, *rrnB3*, DE*lacZ4787*, *hsdR514*, DE(*araBAD*)*567*, *E*(*rhaBAD*)*568*, *rph-1*, *att-lambda*::*pAE12-*del(*oriR6K-cat*::*frt5*), DE(*endA*)::*frt*, *uidA*(*delMluI*)::*pir*(wt), *attHK*::pJK1006-*del1/2* (del*oriR6K-cat*::*frt5,* del*trfA*::*frt*) | William Metcalf, University of Illinois |
| BL21 (DE3) | F^–^ *ompT hsdR17(r_B_^-^ m_B_^+^ ) gal dcm*(DE3) F^–^, *ompT*, *hsdS*_B_(r_B_^–^ m_B_^–^), *gal*(λcI 857, *ind*1, *Sam*7, *nin*5, *lacUV*5-T7*gene*1), *dcm*(DE3) | Novagen |
|  |  |  |
| *Shewanella oneidensis* |  |  |
| MR-1 | Wild type | ATCC |
| ∆*arcS* | SO_0577 (*arcS*) disrupted | This study |
| ∆PFL | SO_2912 (*pflB*) disrupted | This study |
| ∆PDH | SO_0424 (*aceE*) disrupted | This study |
| ∆NDH | SO_1017 (*nuoF*), SO_3517 (*ndh*), SO_0907 (*nqrF-1*), and SO_1108 (*nqrF-2*) disrupted | This study |
| Plasmid |  |  |
| pSMV-10 | 9.1 kb mobilizable suicide vector; *oriR6K*, *mobRP4*, s*acB*, Km^r^, Gm^r^  pSMV-0624 | Chad Saltikov, California Institute of Technology |
| pSMV-arcS | pSMV10 containing the 1.5 kb fusion PCR fragment for *arcS* disruption | This study |
| pSMV-pflB | pSMV10 containing the 1.5 kb fusion PCR fragment for *pflB* disruption | This study |
| pSMV-aceE | pSMV10 containing the 1.5 kb fusion PCR fragment for *aceE* disruption | This study |
| pSMV-nuoF | pSMV10 containing the 1.5 kb fusion PCR fragment for *nuoF* disruption | This study |
| pSMV-ndh | pSMV10 containing the 1.5 kb fusion PCR fragment for *ndh* disruption | This study |
| pSMV-nqrF-1 | pSMV10 containing the 1.5 kb fusion PCR fragment for *nqrF-1* disruption | This study |
| pSMV-nqrF-2 | pSMV10 containing the 1.5 kb fusion PCR fragment for *nqrF-2* disruption | This study |
| pET-28a(+) | Expression vector, T7 promoter | Novagen |
| pET-arcA | pET-28a(+)-based plasmid expressing *N-his-arcA* | This study |

**Supplementary Table 2** Primers used in this study.

| Primer | Sequence (5′–3′) | **Modification,** for use |
| --- | --- | --- |
| qRT-*nuoI*-F | TTTCGAGATGGGCGAGTATC | qRT-PCR for *nuoI* |
| qRT-*nuoI-*R | CGCTCATGCGATAGAAGTTG | qRT-PCR for *nuoI* |
| qRT-*dld-II*-F | CATCGGCACTCAACTTCTCA | qRT-PCR for *dld-II* |
| qRT-*dld-II*-R | CGCAGGTATCAATCACATCG | qRT-PCR for *dld-II* |
| qRT-SO_4509-F | CAAGCCGTTTTGATCAAGGT | qRT-PCR for SO_4509 |
| qRT-SO_4509-R | ATCCTTCTGTGCGATCTTGG | qRT-PCR for SO_4509 |
| qRT-SO_0939-F | AATCGACGCCAAGCATTAAC | qRT-PCR for SO_0939 |
| qRT-SO_0939-R | TTTTCCCCGAGTGCTAATTG | qRT-PCR for SO_0939 |
| qRT-*atpG*-F | CGTGAAAAGCTGGAAAGAGC | qRT-PCR for *atpG* |
| qRT-*atpG*-R | TGCTTGTGCCGATACTTGTC | qRT-PCR for *atpG* |
| qRT-SO_1538-F | ACCTCAGTGATCTTGGCCTC | qRT-PCR for SO_1538 |
| qRT-SO_1538-R | CGCCTTCTTCAATCACAGCA | qRT-PCR for SO_1538 |
| qRT-SO_4360-F | CGTTCATCACACCCGCTAAC | qRT-PCR for SO_4360 |
| qRT-SO_4360-R | CGCATGGCAGGTATAACAGG | qRT-PCR for SO_4360 |
| *arcA*_NdeI_F | CGC**CATATG**CAAAATCCGCACATTCTGATCG | **NdeI,** pET-arcA construction |
| *arcA*_BamHI_R | CGC**GGATCC**TTAGTCTTCTAAGTTACCGCAGAAACG | **NdeI,** pET-arcA construction |
| *nuoA-*gelshift-F | GGTCACGATTAAGTTTCATCC | *nuoA* probe, EMSA |
| Cy3-*nuoA*-gelshift-R | GCAATACATTGGCAACCA | 5'-Cy3, *nuoA* probe, EMSA |
| SO_0011-gelshift-F | GGTATAATCGGGGAGTTTTTA | SO_0011 probe, EMSA |
| Cy3-SO_0011-gelshift-R | TTCTCTGACATATTATTCTCTC | 5'-Cy3, SO_0011 probe, EMSA |
| *sucA*-gelshift-F | GCGAGCTGTGTAATGCAAGAA | SO_1930 probe, EMSA |
| Cy3-*sucA-*gelshift-R | TGATGCCTTGGTGCATTTCTA | 5'-Cy3, SO_1930 probe, EMSA |
| *atpI*-gelshift-F | TGGCTGAATTAGACGGAATTC | *atpI* probe, EMSA |
| Cy3-*atpI*-gelshift-R | TACTCAACTCATCTTCTCCGC | 5'-Cy3, *atpI* probe, EMSA |
| *arcS*_F-O | AAAGATGATGCCTTGGCTGG | *arcS* disruption |
| *arcS*_5-O-SpeI | GATG**ACTAGT**GCTACACAAGAACGATGTG | **SpeI***, arcS* disruption |
| *arcS*_5-I | CTGATCGGTGCAAAAGTTGTCGATCTGCAATACGTG | *arcS* disruption |
| *arcS*_3-I | AACTTTTGCACCGATCAGCACCGTTAATTGTTCGCC | *arcS* disruption |
| *arcS*_3-O-SpeI | TAGC**ACTAGT**CGACATGAATGTACCGTCAG | **SpeI***, arcS* disruption |
| *arcS*_R-O | TTGATACCCATCCTCTGGCA | *arcS* disruption |
| *arcS*_5-linker | CTGATCGGTGCAAAAGTT | *arcS* disruption |
| *arcS*_3-linker | AACTTTTGCACCGATCAG | *arcS* disruption |
| *pflB*_5-O | AGCGTGATGCTATTCACAGGG | *pflB* disruption |
| *pflB*_5-linker | GGTCACACCAGCACCTGA | *pflB* disruption |
| *pflB*_3-O | CATAGTTTCGCGGCTGGGAGG | *pflB* disruption |
| *pflB*_3-linker | TCAGGTGCTGGTGTGACC | *pflB* disruption |
| *pflB*_FO | GCTAGCTGTGATGCAGAG | *pflB* disruption |
| *pflB*_RO | CCATGGGCATACATTGCC | *pflB* disruption |
| *pflB*_5-O-SpeI-IN | GAAGGTAG**ACTAGT**ATCGTCGTTCCGTGCCTG | **SpeI***, pflB* disruption |
| *pflB*_3-O-SpeI-IN | GAAGGTAG**ACTAGT**CACCGTCGAGTTGATAGC | **SpeI***, pflB* disruption |
| *pflB*_5-I | GGTCACACCAGCACCTGAAGATTTCCAATCACCAGG | *pflB* disruption |
| *pflB*_3-I | TCAGGTGCTGGTGTGACCGATCACGCGTACATTCAC | *pflB* disruption |
| *aceE*_5-O | CTGATGTGATCATGGCGC | *aceE* disruption |
| *aceE*_5-linker | GGTGTTAGCCACTGAAGC | *aceE* disruption |
| *aceE*_3-O | GCACTGATCATATCGCCC | *aceE* disruption |
| *aceE*_3-linker | GCTTCAGTGGCTAACACC | *aceE* disruption |
| *aceE*_FO | CGCCAAATGGCCTATAGC | *aceE* disruption |
| *aceE*_RO | CAACGACAGCAGTTTGCC | *aceE* disruption |
| *aceE*_5-O-SpeI-IN | GAAGGTAG**ACTAGT**AGCAAATGATCCTCGAGG | **SpeI***, aceE* disruption |
| *aceE*_3-O-SpeI-IN | GAAGGTAG**ACTAGT**CGATCACATCGACATTGC | **SpeI***, aceE* disruption |
| *aceE*_5-I | GGTGTTAGCCACTGAAGCGGATCTACGTCTTGTAGC | *aceE* disruption |
| *aceE*_3-I | GCTTCAGTGGCTAACACCGATCAATCCACAGTACGC | *aceE* disruption |
| *nuoF*_3-O-SpeI-IN | GAAGGTAG**ACTAGT**CAAACTGCATGTCCCACT | **SpeI***, nuoF* disruption |
| *nuoF*_3-O | GCATTGCTGGCAGATGCT | *nuoF* disruption |
| *nuoF*_5-O-SpeI-IN | GAAGGTAG**ACTAGT**TGATCAAGGGCAGCATGG | **SpeI***, nuoF* disruption |
| *nuoF*_5-O | CTTTGCTCACCTGCAACA | *nuoF* disruption |
| *nuoF*_FO | GTTACTACCTGACCAGCG | *nuoF* disruption |
| *nuoF*_RO | GCCGTGGAAACGGTTTTC | *nuoF* disruption |
| *nuoF*_3-I | GGTTCAGTGCAGGCTGGTGATCCAGCCCAATCTGCT | *nuoF* disruption |
| *nuoF*_5-I | ACCAGCCTGCACTGAACCGAGAACTTTGCTCTGTGG | *nuoF* disruption |

**Supplementary Table 2** Continued.

| Primer | Sequence (5′–3′) | **Modification,** for use |
| --- | --- | --- |
| *nuoF*_5-linker | ACCAGCCTGCACTGAACC | *nuoF* disruption |
| *nuoF*_3-linker | GGTTCAGTGCAGGCTGGT | *nuoF* disruption |
| *ndh*_3-linker | GCTGGTTCAGTGACCGCT | *ndh* disruption |
| *ndh*_5-linker | AGCGGTCACTGAACCAGC | *ndh* disruption |
| *ndh*_FO | TAGCGCCTTCATTTTCGG | *ndh* disruption |
| *ndh*_RO | TGAGCTCGTTATTCACCC | *ndh* disruption |
| *ndh*_3-O | ACCGCCACTTTCAGAAGATCC | *ndh* disruption |
| *ndh*_5-O | GCGCTAACAATGTGTAAACGG | *ndh* disruption |
| *ndh*_3-I | GCTGGTTCAGTGACCGCTGGCCAAAGCTGAAATTAC | *ndh* disruption |
| *ndh*_5-I | AGCGGTCACTGAACCAGCCACTATTCGCTTAGTAGC | *ndh* disruption |
| *ndh*_3-O-SpeI-IN | GAAGGTAG**ACTAGT**GCGAGGGGATTATAGAGC | **SpeI***, ndh* disruption |
| *ndh*_5-O-SpeI-IN | GAAGGTAG**ACTAGT**ATCGCATCAAGCCAAACG | **SpeI***, ndh* disruption |
| *nqrF-1*_3-linker | GGTGCTGTGTCAACCGCT | *nqrF-1* disruption |
| *nqrF-1*_5-linker | AGCGGTTGACACAGCACC | *nqrF-1* disruption |
| *nqrF-1*_FO | TGTGCGTGAGTTGTTAGG | *nqrF-1* disruption |
| *nqrF-1*_RO | AAGAATGCCACGGTTAGC | *nqrF-1* disruption |
| *nqrF-1*_3-O | GTAGTGAGATCAATGCGGCAC | *nqrF-1* disruption |
| *nqrF-1*_5-O | GGCTGGTACTTACCTAACGAG | *nqrF-1* disruption |
| *nqrF-1*_3-I | GGTGCTGTGTCAACCGCTCGGTGATTAAGATGCTCG | *nqrF-1* disruption |
| *nqrF-1*_5-I | AGCGGTTGACACAGCACCATGCCTATGCCTATTGCC | *nqrF-1* disruption |
| *nqrF-1*_3-O-SpeI-IN | GAAGGTAG**ACTAGT**CCTAACTACAACACAGGG | **SpeI***, nqrF-1* disruption |
| *nqrF-1*_5-O-SpeI-IN | GAAGGTAG**ACTAGT**GCATTAACGTGATCCAGC | **SpeI***, nqrF-1* disruption |
| *nqrF-2*_3-linker | AACTCAATCGTGGCTCAG | *nqrF-2* disruption |
| *nqrF-2*_5-linker | CTGAGCCACGATTGAGTT | *nqrF-2* disruption |
| *nqrF-2*_FO | GGATGGTATTGGTAACGG | *nqrF-2* disruption |
| *nqrF-2*_RO | CATAGACATAGTGCCAGG | *nqrF-2* disruption |
| *nqrF-2*_3-O | ATCGGTCGGCTTCTCTATTGC | *nqrF-2* disruption |
| *nqrF-2*_5-O | GGTGTTCAAATCCTGCACAAG | *nqrF-2* disruption |
| *nqrF-2*_3-I | AACTCAATCGTGGCTCAGCCTGTTGGATGACTTCGG | *nqrF-2* disruption |
| *nqrF-2*_5-I | CTGAGCCACGATTGAGTTGGTAAACCTCAAGCGGAG | *nqrF-2* disruption |
| *nqrF-2*_3-O-SpeI-IN | GAAGGTAG**ACTAGT**TAGCATCGCCTTTAGTGC | **SpeI***, nqrF-2* disruption |
| *nqrF-2*_5-O-SpeI-IN | GAAGGTAG**ACTAGT**CTGGTATCAACCAAACGG | **SpeI***, nqrF-2* disruption |
