# Supplementary material for: Electrochemically active bacteria sense electrode potentials for regulating catabolic pathways
Source: Nat Commun. 2018 Mar 14;9:1083. doi: 10.1038/s41467-018-03416-4 (PMC5852097; doi:10.1038/s41467-018-03416-4)
Supplement: Supplementary file 3 — Description of Additional Supplementary Files [file 41467_2018_3416_MOESM3_ESM.pdf]

Descriptions of Additional Supplementary Files:

File Name: Supplementary Data 1
Description: List of all electrode potential-responsive genes in S. oneidensis MR-1

File Name: Supplementary Data 2
Description: List of electrode potential-responsive genes found in ΔarcS

File Name: Supplementary Data 3
Description: List of electrode potential-responsive genes found in both WT and Δ*arcS*
